# Soy Consumption and the Risk of Type 2 Diabetes and Cardiovascular Diseases: A Systematic Review and Meta-Analysis

**DOI:** 10.3390/nu15061358

**Published:** 2023-03-10

**Authors:** Xinrong Zuo, Rui Zhao, Minming Wu, Qianyi Wan, Tao Li

**Affiliations:** 1 Department of Anesthesiology, The Affiliated Hospital of Southwest Medical University, Luzhou 646000, China; 2 Department of Anesthesiology, Laboratory of Mitochondria and Metabolism, National Clinical Research Center for Geriatrics, West China Hospital of Sichuan University, Chengdu 610041, China; 3 Division of Gastrointestinal Surgery, West China Hospital of Sichuan University, Chengdu 610041, China

**Keywords:** soy, health, type 2 diabetes, cardiovascular diseases, meta-analysis

## Abstract

Soy is rich in plant protein, isoflavones, and polyunsaturated fatty acids. To clarify the associations between soy intake and type 2 diabetes (T2D) and cardiovascular diseases (CVDs) events, we performed a meta-analysis and review. A total of 1963 studies met the inclusion criteria, and 29 articles with 16,521 T2D and 54,213 CVDs events were identified by the eligibility criteria. During a follow-up of 2.5–24 years, the risk of T2D, CVDs, coronary heart disease, and stroke in participants with the highest soy consumption decreased by 17% (total relative risk (TRR) = 0.83, 95% CI: 0.74–0.93), 13% (TRR = 0.87, 95% CI: 0.81–0.94), 21% (TRR = 0.79, 95% CI: 0.71–0.88), and 12% (TRR = 0.88, 95% CI: 0.79–0.99), respectively, compared to the lowest sot consumption. A daily intake of 26.7 g of tofu reduced CVDs risk by 18% (TRR = 0.82, 95% CI: 0.74–0.92) and 11.1 g of natto lowered the risk of CVDs by 17% (TRR = 0.83, 95% CI: 0.78–0.89), especially stroke. This meta-analysis demonstrated that soy consumption was negatively associated with the risks of T2D and CVDs and a specific quantity of soy products was the most beneficial for the prevention of T2D and CVDs. This study has been registered on PROSPERO (registration number: CRD42022360504).

## 1. Introduction

Soy is a major source of plant protein, dietary fiber, polyunsaturated fatty acids, lecithin, stigmasterol and isoflavones. Soy protein is the only plant-derived complete protein and it accounts for 35–40% of soy, containing all the essential amino acids in animal proteins [1]. Its amino acid composition is similar to human essential amino acid composition, and its content is sufficient [1]. Animal protein is rich in a variety of essential amino acids, but also rich in fat and cholesterol, leading to a high incidence of various chronic diseases [2]. Additionally, soy protein does not contain cholesterol, and the contents of methionine and branched-chain amino acids (BCAAs) are low compared with animal proteins [3,4]. Although leucine or its metabolite β-hydroxy-β-methylbutyric acid can improve muscle function [5], aberrant metabolism of BCAAs and high circulating concentrations of BCAAs are a hallmark of metabolic disorders, including obesity, insulin resistance and type 2 diabetes mellitus, cancer, and heart failure [6,7]. Studies have found that a low BCAA diet can increase the survival rate of premature aging in mice, delay frailty, and promote metabolic health [8]. Restricting dietary BCAAs can increase the health span and life span of mice, which is a potential transformable intervention for the promotion of healthy aging [8]. Polyunsaturated fatty acids, lecithin, and stigmasterol are also the main components of soy. Studies have reported that these ingredients can reduce the total cholesterol and low-density lipoprotein cholesterol levels, improve diabetes and protect cardiovascular health [9,10]. Soy foods are classified into fermented and non-fermented soy foods. Through Bacillus subtilis and aspergillus fermentation, the nutritional value of fermented soy products is enhanced, improving the digestibility, and increasing the soy protein and isoflavone profiles compared to non-fermented soy foods [11]. The health benefits of fermented soy foods includes antioxidant, anticancer, anti-inflammatory, anti-hyperlipidemic, and the prevention of osteoporosis [11]. Isoflavones in non-fermented foods, mainly soy milk and tofu, can be destroyed in the process of processing and cooking. Thus, non-fermented soy products might lower the protective effects of soy on cardiovascular and chronic disease health [12,13].

Cardiovascular diseases (CVDs) remain a major cause of mortality and disability worldwide, accounting for one-third of global deaths [14]. CVDs mortality increased by 34.9% from 1990 to 2019 based on the Global Burden of Disease Study [15]. Type 2 diabetes (T2D) has been the ninth major cause of death worldwide, following a significant increase of 70% since 2000 [15], which reduces lifespan and has an enormous economic and social psychological burden on a global scale [16]. Meanwhile, T2D is globally considered one of the most common metabolic complications of CVDs, contributing to 10.2% [15]. Thus, T2D and CVDs have common risk factors such as obesity, a lack of activity, an unhealthy diet and lifestyle, and an aging population [14,16]. Studies have demonstrated that a healthy diet and lifestyle could lower the incidence of T2D and CVDs [2,16,17,18,19,20]. Evidence has further revealed that a higher plant protein intake reduced CVDs mortality and improved metabolic health [2,21]. Plant protein may also have antioxidant, anti-inflammatory, antihypertensive, and antibacterial activity [1,2,22]. Soy is rich in plant proteins, so the exploration of soy consumption related to T2D and CVDs will be of great importance for the prevention of T2D and CVDs strategies.

An increasing number of observational studies and meta-analyses have been focusing on the relationship between increased soy consumption and the risk of T2D and CVDs, but the conclusions were inconsistent [23,24] To resolve these inconsistencies, we included more databases and the latest literature to evaluate the association between soy consumption and the risk of T2D and cardiovascular events.

## 2. Materials and Methods

This systematic review and meta-analysis was reported based on the Preferred Reporting Items for Systematic Reviews and Meta-Analyses (PRISMA) guideline [25]. We also adhered to the Meta-analysis of Observational Studies in Epidemiology (MOOSE) reporting guideline [26].

### 2.1. Data Sources and Search Strategy

Three reviewers conducted a systematic search of all articles published up to 14 October 2022, of online databases, including Medline, PubMed, Web of Science core collection, and Embase. The research was restricted to human studies. Detailed search strategies can be found in Table S1. To avoid missing any publications, we also evaluated and checked all studies in the prior reviews and contacted the author of the original study.

### 2.2. Eligibility Criteria and Study Selection

Studies were included in this meta-analysis according to the following criteria: (1) the study design was a case–control or nested case–control or cohort, (2) the exposure factor was soybean, soy food, or soy protein consumption, (3) the outcome of interest was population aged more than 18 years with T2D or CVDs events, including coronary heart disease (CHD) and stroke, and those who reported odds ratio (OR), relative risk (RR), or hazard ratio (HR) associated with 95% confidence interval (95% CI) between the interested exposure and outcome. Exclusion criteria were as follows: (1) Case reports, editorials, letters to editors, comments, conference abstracts, short communications, reviews, cross-sectional, and animal studies were not considered. (2) If study participants were reported more than once, we selected the greatest number of cases or the result with a longer follow-up time. Three independent reviewers first selected relevant studies by screening the titles and then, reviewed the full texts according to the inclusion/exclusion criteria to decide the final relevant references to be included in the review. Any discrepancy between the three co-authors was resolved with a fourth author.

### 2.3. Data Extraction

Three investigators used a predefined form to extract information, including first author, publication year, database used, country, study design, sample size, duration of follow-up in cohort studies, type of Food Frequency Questionnaire (FFQ), exposure assessment, ascertainment of outcomes (CHD, stroke, or total CVDs), comparison, adjustment for confounding covariates, and the Newcastle–Ottawa Scale (NOS) [27] score. We extracted HR, RR, or OR those that reflected the maximum extent of adjustment for variables. Any results stratified by sex or endpoints were treated as two separate reports. We have tried to contact the authors to obtain some available research statistics, but we have not yet received a response.

### 2.4. Quality Assessment

The methodological quality of each study was independently evaluated by three reviewers using NOS [27]. This scale consists of three parameters of quality: four stars for the selection of participants, two stars for comparability, and three stars for assessment of exposure (for case–control studies) or outcomes (for cohort studies). A maximum of nine stars can be given to each study. In our systematic review, those that achieved a score of 0–4, 5–7, or 8 or more, were, respectively, considered to be low, moderate, and high-quality publications. Disagreements were resolved by discussing with a fourth author.

### 2.5. Statistical Analysis

Review Manager, version 5.3 (The Nordic Cochrane Centre, The Cochrane Collaboration, Copenhagen, Denmark) and Stata, Version 14.0 (Stata Corp, College Station, TX, USA) were used to perform all statistical analyses. A random-effects model was used to calculate total relative risk (TRR) and 95% CI for the highest category compared to the lowest category analysis of each study in this meta-analysis. Forest plot was created to visually assess the pooled result for each study and overall estimate. Based on the statistical test of *I*^2^, the Q-test was used to estimate the heterogeneity between studies. A *p* value less than 0.10 or an *I*^2^ value greater than 50% indicated statistical heterogeneity. Meta-regression and subgroup analyses were used to analyze sources of heterogeneity. Sensitivity analyses were performed by excluding each study to assess the stability of the study. Egger’s test and Begg’s test were designed to assess publication bias.

Methods described by Greenland [28] and Orsini [29] were carried out to calculate the dose–response analysis between soy foods consumption and T2D/CVDs events. A two-stage random-effects dose–response meta-analysis was applied to examine a probable non-linear association between intakes of soy foods and T2D/CVDs events. A two-stage generalized least squares trend estimation method was employed to explore linear dose–response relationships. *p* values were considered significant at the level < 0.05.

## 3. Results

### 3.1. Literature Search Results

**Figure 1:**
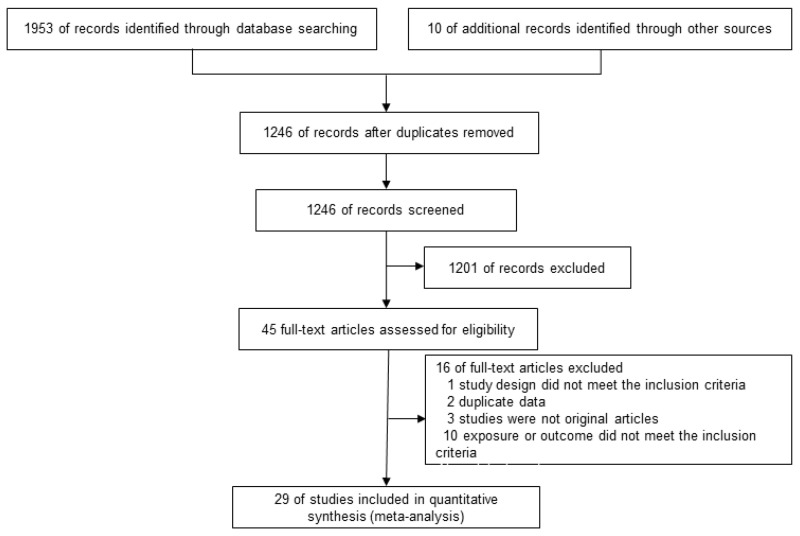
The flow chart of the literature selection.

This study included 45 articles by screening the titles and abstracts and sixteen articles were further excluded based on the inclusion and exclusion criteria (Table S2 in online supplementary materials). Finally, we identified 9 articles that were related to T2D [30,31,32,33,34,35,36,37,38] and 20 articles involving CVDs [12,13,39,40,41,42,43,44,45,46,47,48,49,50,51,52,53,54,55,56,57]. All the studies were published between 2001 and 2021. Among the 29 studies, 23 researchers used a validated semiquantitative FFQ to collect dietary information. One study used a non-validated semiquantitative FFQ and four studies were unclear. Only one study used validated weight and record all foods and beverages on 3 consecutive days. The countries where the studies were conducted are as follows: three were conducted in the USA, twenty-six in Asia, thirteen in Japan, nine in China, two in Korea, one in Iran, and one in Vietnam. The systematic literature search flowchart was shown in Figure 1 according to PRISM.

### 3.2. Study Characteristics and Quality Assessment

The main characteristics of the included studies were given in Table 1. All the 29 studies selected for quantitative analysis consisted of 1,660,304 participants during the follow-up of 2.5–24 years, including 16,521 T2D events (morbidity) and 54,213 CVDs events (morbidity and mortality), which were composed of 22,112 stroke events, 12,906 CHD events, and 19,195 other CVDs events. Based on the NOS, all the quality scores ranged from 5–9 and 19 studies were of high quality (Table S3).

### 3.3. Synthesis with Meta-Analysis

#### 3.3.1. Association between Soy Consumption and the Risk of T2D and CVDs

**Figure 2:**
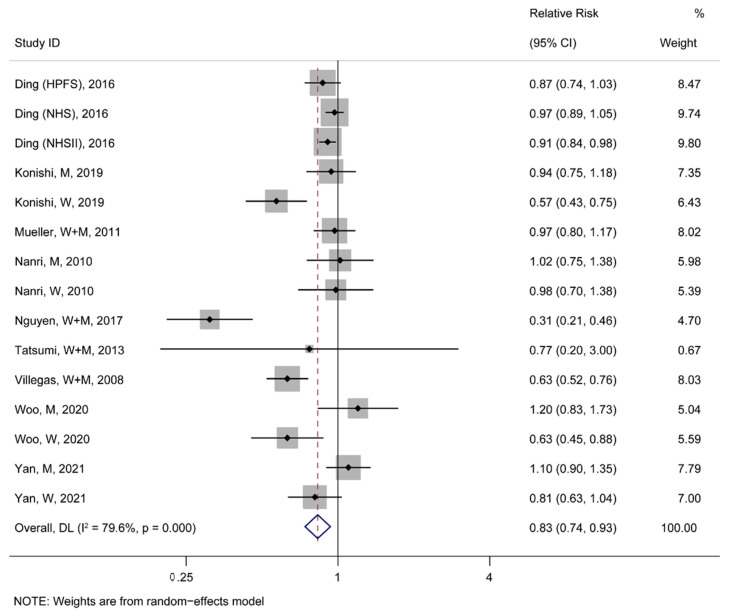
The pooled risk association of T2D with soy consumption [30,31,32,33,34,35,36,37,38]. In the forest plot, the black point represents the point estimate of the effect size of each study, the gray square means the weight of each study, the line length represents the 95% confidence interval (CI) of the effect size of each study, the diamond represents the meta-analysis synthesizes the summary results of each study, the diamond center represents the point estimate of the effect size of the summary results, and the diamond width represents the 95% CI of the effect size of the summary results.

**Figure 3:**
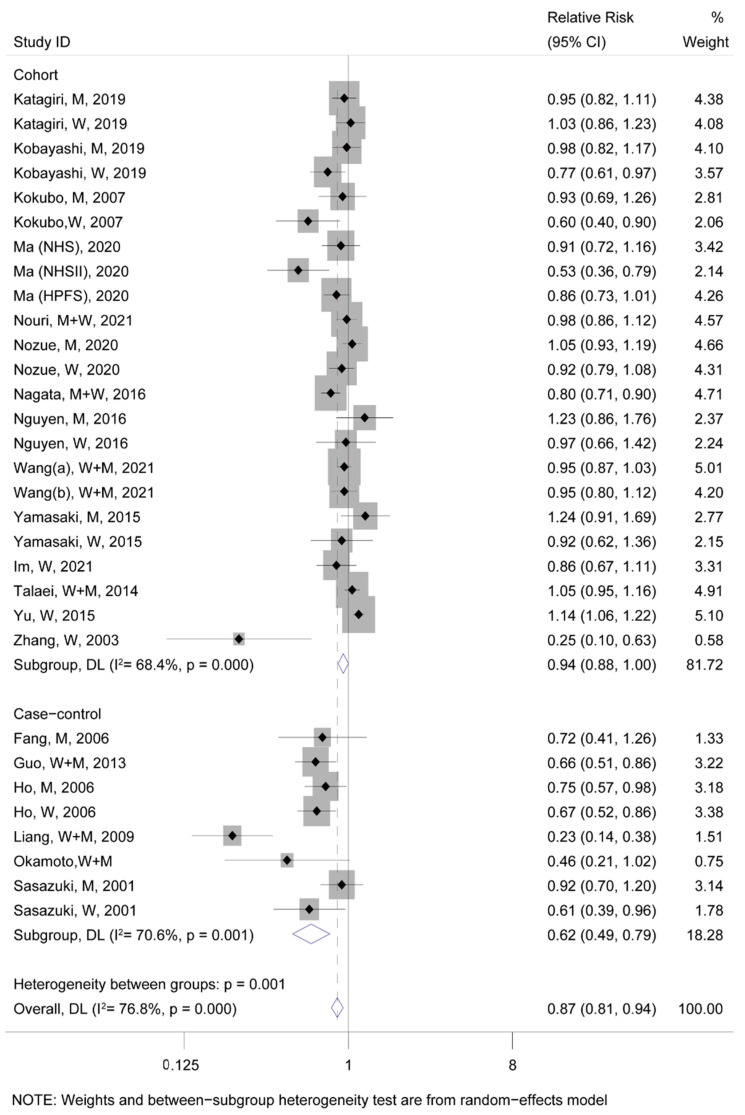
The pooled risk association of CVDs with soy consumption. In the forest plot, the black point represents the point estimate of the effect size of each study, the gray square means the weight of each study, the line length represents the 95% CI of the effect size of each study, the diamond represents the meta-analysis synthesizes the summary results of each study, the diamond center represents the point estimate of the effect size of the summary results, and the diamond width represents the 95% CI of the effect size of the summary results [12,13,14,39,41,42,43,44,46,48,49,50,51,52,53,54,55,56,57].

A meta-analysis for fifteen studies from nine articles about T2D events demonstrated a significant negative association between soy consumption and the incidence rate of T2D (TRR = 0.83, 95% CI: 0.74–0.93; Figure 2). Moreover, eight case–control and twenty-three cohort studies about CVDs events were analyzed together, revealing that the highest soy consumption could abate the incidence rate of CVDs events by 13% compared to the lowest (TRR = 0.87, 95% CI: 0.81–0.94; Figure 3). Additionally, it was found that the highest soy consumption reduced the incidence rate of CHD events by 21% (TRR = 0.79, 95% CI: 0.71–0.88; Figure S1) and stroke events by 12% (TRR = 0.88, 95% CI: 0.79–0.99; Figure S2) compared to the lowest soy consumption, respectively.

#### 3.3.2. Subgroup Analysis between Soy Foods Consumption and the Risk of T2D and CVDs Events

To investigate the association of specific soy products with T2D and CVDs, we performed a subgroup analysis. All subgroup analyses of soy foods were shown in Table 2. We found that non-fermented and fermented soy food were not associated with T2D events (*p* > 0.05). However, dried and other soy foods were significantly and negatively associated with T2D events (TRR = 0.75, 95% CI: 0.61–0.93). In cardiovascular events, only tofu (TRR = 0.89, 95% CI: 0.80–0.99) and natto (TRR = 0.82, 95% CI: 0.75–0.90) were negatively associated with CVDs, whereas soy milk (TRR = 0.81, 95% CI: 0.59–1.12) and miso soup (TRR = 0.93, 95% CI: 0.84–1.03) were not. Further analyses found that only tofu was associated negatively with CHD events (TRR = 0.81, 95% CI: 0.67–0.98), and only natto was associated negatively with stroke events (TRR = 0.81, 95% CI: 0.72–0.91).

Through a dose–response analysis, the non-linear significant relationships were found between tofu and CVDs (*P*_nonlinearity_ = 0.003, Figure S3A) and between natto and CVDs (*P*_nonlinearity_ < 0.001, Figure S3B). Meanwhile, a non-linear significant relationship between the natto intake and stroke events was also observed (*P*_nonlinearity_ < 0.001, Figure S3C). A daily intake of 26.7 g of tofu reduced the CVDs risk by 18% (TRR = 0.82, 95% CI: 0.74–0.92). When the intake dose exceeded 63 g, the tofu had no protective effect on CVDs (Figure S3A). A daily intake of 11.1 g of natto lowered the risk of CVDs by 17% (TRR = 0.83, 95% CI: 0.78–0.89), especially stroke. When the daily intake was less than 32 g, natto was beneficial for CVDs (stroke) (Figure S3B,C). Additionally, no significant linear or non-linear associations were found between miso and CVDs (*P*_linearity_ = 0.72, *P*_nonlinearity_ = 0.13; Figure S3D), between natto and CHD (*P*_linearity_ = 0.91, *P*_nonlinearity_ = 0.70; Figure S3E), or between miso and stroke (*P*_linearity_ = 0.62, *P*_nonlinearity_ = 0.19; Figure S3F).

#### 3.3.3. Subgroup Analysis between Baseline Characteristics and the Risk of T2D and CVDs Events

To further investigate the association between soy consumption and T2D and CVDs under different baseline characteristics, we performed the following subgroup analysis. First, a significant negative correlation between soy consumption and T2D in women (TRR = 0.78, 95% CI: 0.67–0.91) was demonstrated, but neither was found in men (TRR = 0.99, 95% CI: 0.89–1.1) nor in both included together (TRR = 0.56, 95% CI: 0.18–1.70; Table S4) by stratifying analysis. It also negatively correlated between soy consumption and CVDs events in women (TRR = 0.80, 95% CI: 0.68–0.94) but not in men (TRR = 0.96, 95% CI: 0.89–1.04; Table S4). Further analysis found that high soy consumption had a low risk of CHD events in women and men but did not reduce the risk of stroke in women or men (Table S4). Second, we found that soy consumption lowered the risk of T2D and CVDs in Asians and Americans (Table S4). Meanwhile, soy consumption reduced the risk of CHD (TRR = 0.79, 95% CI: 0.69–0.90) and stroke (TRR = 0.89, 95% CI: 0.80–0.99) in Asians but did not reduce the risk of CHD (TRR = 0.79, 95% CI: 0.62–1.01) and stroke (TRR = 0.72, 95% CI: 0.41–1.26) in Americans. Third, soy consumption had a negative correlation with T2D, CHD, and CVDs events in case–control and cohort studies (Table S4). However, it was not associated with the risk of stroke events in cohort studies (TRR = 0.96, 95% CI: 0.87–1.05). Fourth, the follow-up time did not affect the relationship between soy consumption and the risk of T2D, CVDs, CHD, and stroke events (Table S4).

### 3.4. Publication Bias

The visual inspection identified that cohort studies of CVDs had publication bias (*P*_Egger’ test_ = 0.18 and *P*_Begg’s test_ = 0.01; Figure S4A), while case–control studies (*P*_Egger’ test_ = 0.15 and *P*_Begg’s test_ = 0.38; Figure S4B) related to CVDs and studies between soy intake and T2D risk (*P*_Egger’ test_ = 0.17 and *P*_Begg’s test_ = 0.32; Figure S4C) did not. The publication bias of high-quality cohort studies might be attributed to some differences in baseline characteristics, such as subtypes of CVDs and long follow-up times. Sensitivity analysis indicated a stable publication bias between soy and T2D, and between soy and CVDs (Figure S5A,B).

## 4. Discussion

### 4.1. Main Findings and Interpretation

Besides updating the data by including more studies, there were three strengths compared to the results of the previous meta-analysis related to soy/soy products and CVDs and T2D [23,24,58,59]. First, the subgroup analysis of soy products clarified the specific relationship between soy products and CVDs and T2D. Second, the dose–response analysis based on the subgroup analysis revealed the specific intake dose of bean products and the risk of CVDs. Third, we comprehensively elaborated on the possible mechanisms of soybean components in protecting T2D and CVDs in the discussion, especially soybean protein peptides, isoflavones, unsaturated fatty acids, lecithin, stigmasterol, and low BCAAs. Meanwhile, we also obtained the consistent conclusion that soy intake was negatively associated with the risk of T2D, CVDs, CHD, and stroke events [23,24,58].

It was known that soy protein and flavonoids were important parts of the active ingredients of soy, which could reduce the risk of CVDs and diabetes through antioxidative and anti-inflammatory properties [1,11]. More clinical and basic studies also supported our conclusion [6,23,59]. Animal studies in diabetic rats and mice revealed that consumption of soy protein peptides, such as β-conglycinin, soymorphin-5, and Vglycin, improved muscle glucose uptake, decreased blood glucose and triglyceride levels, increased insulin sensitivity, and restored pancreatic function with higher plasma adiponectin and phosphorylated adenosine monophosphate-activated protein kinase (AMPK) [60,61]. Evidence showed that isoflavone improved the endothelial function, lowered blood pressure and blood glucose, and had beneficial effects on decreasing the cardiovascular risk of patients with T2D by affecting AMPK phosphorylation and glucose transporter 4 (GLUT4) in vitro and in preclinical and clinical studies [62,63,64].

Interestingly, soy does not contain cholesterol, but its plant components, including lecithin, stigmasterol, soy protein peptides, and isoflavones, improve lipid profiles through different mechanisms. For example, phosphatidylcholine in soybean lecithin may regulate lipid metabolism by dissolving cholesterol in the intestine and reducing intestinal cell absorption [9,65]. Secondly, lecithin decreases blood lipids by inhibiting intestinal cholesterol absorption and promoting cholesterol excretion [9]. Lecithin cholesterol acyl transferases (LCAT) are responsible for reverse cholesterol transport from extra-hepatic tissues back to the liver and the activity of LCAT is closely related to cardiovascular health [66]. In recent years, soybean lecithin has been used as a functional food supplement to improve blood lipids [67]. Stigmasterol, as a common phytosterol in soybeans, has no effect on the apolipoprotein E genotype [68], and the composition of intestinal flora [69] may be involved in regulating its cholesterol-lowering effect. Studies have reported that stigmasterol can improve blood pressure and reduce the risk factors of cardiovascular diseases in postmenopausal women [70]. It can also improve blood glucose and induce β-cell regeneration by improving GLUT4 translocation and insulin resistance [10]. Furthermore, soy proteins and isoflavones also reduced blood cholesterol and triglyceride [71,72], which might be beneficial to cardiovascular health and systemic metabolism. Therefore, the lipid-lowering effect of soybeans may be enhanced and mediated by the synergistic effect of intestinal factors and soy components [9]. In the future, soy protein peptides and isoflavones might emerge as complementary or alternative treatments for diabetes and cardiovascular disease [63]. Soy is rich in unsaturated fatty acids and dietary fiber. Additionally, some research has discovered that unsaturated fatty acids, including n-3 long-chain polyunsaturated fatty acids, and linoleic acid, improved fasting glucose levels and insulin resistance, and reduced the risk of stroke and CHD [72,73,74,75]. Similarly, dietary fiber could increase the flora gene richness and activate acetic acid and butyric acid synthesis pathways to promote insulin secretion and intervene in diabetes by modulating gut microbiota [76,77,78].

Additionally, compared to the protein derived from animals, the protein derived from soy had a low concentration of BCAAs of only 4% [79]. However, with the widespread application of metabolomic profiling, increased levels of BCAAs and related metabolites are now widely considered to be a metabolic hallmark of obesity, insulin resistance, and T2D in humans [80]. Meanwhile, some research has reported a direct role of BCAAs in heart failure (HF), vascular disease, hypertension, and arrhythmias [7]. Epidemiologically, increased plasma BCAA concentrations were biomarkers for HF, C, HD, and hypertension and they could predict adverse outcomes in individuals with CHD and HF, and predict stroke, myocardial infarction (MI), coronary revascularization, and death from cardiovascular causes in individuals without CVDs [7]. Thus, this might be one of the key mechanisms through which soy intake could reduce the risk of T2D and CVDs.

Although fried or other soy intakes were negatively associated with T2D, we should be cautious about the result, since only five pooling studies were included. Our result revealed that the consumption of soy foods, especially tofu and natto, was negatively associated with the risk of T2D and total CVDs, including CHD and stroke. This is because fermented soy foods, such as natto, contain higher peptides, amino acids, and other breakdown products and consequently enhanced the antioxidant capacity [11]. Additionally, angiotensin-converting enzyme (ACE) inhibitory peptides, richen in natto, blocked ACE and modulated the rennin–angiotensin system to regulate blood pressure [1]. Natto is a salt-free fermented bean product compared to miso soup. Therefore, natto, but not miso soup was associated with stroke and total CVDs events [1]. The processing of tofu and natto could well reduce the fat content of soy, which might be beneficial in decreasing the incidence of T2D and CVDs [81].

The results of the subgroup analysis by gender showed a significant negative relationship between soy intake with T2D and CVDs in women but not in men. Soy intake was negatively associated with CHD and women had a lower risk than men, whereas it was not associated with stroke in men and women. Such a difference could be attributed to isoflavones. Soybean isoflavones are phytoestrogens that are bound to estrogen receptors in different tissues to exert a two-way regulatory effect on endogenous estrogen and maintain the balance of estrogen levels in women [1]. Isoflavones exerted cardioprotective functions and a protective role of soy against the development of T2D by estrogen-like effect and antioxidation [30,42]. However, soy or isoflavones have no effect on testosterone or estrogen levels in men [72]. In addition, the protective effect of unsaturated fatty acids on CVDs in women was more significant than that in men [75,82]. Through further analysis of the correlation between gender and stroke, we found that men generally had a higher risk of stroke than women, excluding Yu’s research. When we excluded the study, the occurrence of stroke in women was significantly negatively correlated, but this was not the case in men. The discrepant differences from these studies may be partly explained by differences in the type and amount of soy products consumed, population characteristics, and variations in bioactive peptides of soy protein and isoflavone metabolism [54].

Results from our subgroup analysis according to the study regions showed a more significant negative relationship between soy intake and CHD and stroke in Asians and Americans. Since only three cohort studies in the United States were summarized, we should be cautious about the protective effect of soy intake on CVDs in Americans. There are more studies from Asians, and the difference could be attributed to the much higher intakes of soy in Asians than in Westerners and different ethnicities, local gastronomy, food habits, cooking style, and processing of soy. Soy consumption was traditionally common and had become mainstream among Asians [72,83].

Results from summarizing case–control and cohort studies showed protective effects of soy foods on the risk of total CVDs and CHD, whereas there were no protective effects between soy foods and stroke in the meta-analysis of cohort studies. Cohort studies included a larger number of population; dietary exposure was first determined and CVDs were detected after a period of follow-up. However, case–control studies may be attributed to selection and recall bias, because the exposure information of dietary intake was reported after the diagnosis of CVDs was determined. Therefore, the overall results of reducing the risk of CVDs, stroke, and CHD should not be overemphasized in case–control studies.

### 4.2. Strengths and Limitations

There are several strengths to this study. First, we conducted a comprehensive literature search for all the available evidence from existing cohorts and case–control studies and performed a dose–response analysis to investigate the associations of soy foods intake with T2D and CVDs events. Second, an extensive subgroup analysis and meta-regression were conducted to explore the potential sources of heterogeneity and their influence on pooled risk estimates.

Several limitations should be considered. First, although we conducted a systematic literature search and included several eligible studies, the dose-analysis was incomplete due to a lack of essential data. Additionally, the exposure medians for individual studies in the dose–response analysis were approximately estimated and some errors might exist. Second, heterogeneity could be due to the diet habits of different populations, such as varieties and components of soy products, and different intake levels and frequencies of soy foods. Although we carried out stratified analyses using the random effects model, the evident heterogeneity still existed, which was caused by sociodemographic risk factors for T2D and CVDs, and other risk factors. Third, assessing the quality of evidence using the NOS scale might lead to subjectivity.

## 5. Conclusions

This systematic review and meta-analysis found that soy intake was negatively associated with T2D and CVDs (including CHD and stroke). The daily intake of 26.7 g of tofu reduced CVDs risk by 18%. A daily intake of 11.1 g of natto lowered the risk of CVDs by 17%, especially stroke. Our findings may support a specific intake of tofu and natto for preventing CVDs and providing greater longevity. More prospective and multi-center studies should be performed to find the protective association between dose-analysis of soy foods and T2D and CVDs.

## Figures and Tables

**Table 1:** Basic characteristics of the included prospective and case–cohort studies by exposures reported.

First Author (year)	Cohort Name	Country	Sample Size	Follow-up (Median, y)	Dietary Assessment	Exposure Assessment	Outcome (n)	Comparison	Adjustments	NOS Score
*Prospective cohort studies*										
Yamasaki (2015) [53]	JMS	Japan	11,066	11.8	Self-administered, FFQ, 30-item, and validated	Soy	CVDs deaths (198)	Almost daily vs. 1–2 times/week	a and b	8
Yamasaki (2015) [53]	JMS	Japan	11,066	11.8	Self-administered, FFQ, 30-item, and validated	Soy products	CVDs deaths (217)	Almost daily vs. 1–4 times per week	a and b	8
Nagata (2016) [48]	Takayama study	Japan	29,079	16	Self-administered, FFQ-169, and validated	Natto	CVDs deaths (1678), stroke (677), and CHD (308)	7.3 vs. 0 g/d	a, b, and c	9
Zhang (2003) [57]	SWHS	China	64,915	2.5	Interview, FFQ, and validated	Total soy protein	CHD (62)	Q4 vs. Q1 ≥ 11.19 vs. <4.5 g/d	a, b, and c	8
Yan (2021) [31]	JACC Study	Japan	21,925	5	Self-administered, FFQ-40, and validated	Tofu	T2D (593)	Almost daily vs. <3 times/week	a, b, and c	9
Yan (2021) [31]	JACC Study	Japan	21,925	5	Self-administered, FFQ-40, and validated	Miso soup	T2D (593)	≥3 bowls/day vs. <1 bowl/day	a, b, and c	9
Woo (2020) [35]	MR Cohort	Korea	8269	6.18	Interview, FFQ-106, and validated	Soy protein	T2D (531)	Q4 vs. Q1	a, b, and c	9
Wang-a (2021) [12]	CKB study	China	487,034	8.2	Interview, FFQ, and validated	Soy	CVDs deaths (12582), CHD (3764), and stroke (5916)	≥4 days per week vs. never or rarely	a, b, and c	7
Wang-b (2021) [12]	CKB study	China	22,923	7.8	Interview, FFQ, and validated	soy	CVDs deaths (2860), CHD (1123), and stroke (1473)	≥4 days per week vs. never or rarely	a, b, and c	7
Villegas (2008) [30]	SWHS	China	64,191	4.6	Interview, FFQ-77, and validated	Soybeans	T2D (1605)	Q5 vs. Q1 32 vs. 2.8 g/d	a, b, and c	9
Tatsumi (2013) [34]	The Saku Study	Japan	3039	4	Self-administered, FFQ, and non-validated	soybean products	T2D (204)	≥4 times/week vs. 0–1 time/week	a, b, and c	7
Talaei (2014) [52]	SCHS	China	60,298	14.7	Interview, FFQ-165, and validated	Tofu equivalents	CVDs deaths (4780), CHD (2697), and stroke (1298)	Q4 vs. Q1 197 vs. 42.8 g/d	a, b, and c	9
Nozue (2020) [51]	JPHC I, II	Japan	79,648	13.3	Self-administered, FFQ-138, and validated	Total soy products	CVDs events (4427), stroke (3743), and CHD (684)	Q4 vs. Q1 140.9 vs. 33.6 g/d	a, b, and c	8
Nouri (2021) [50]	ICS	Iran	5432	13	Interview, FFQ-48, and validated	Soybeans	CVDs events (751)	≥1 times /week vs. <1 times/week	a and b	7
Ma (2020) [13]	NHS, NHSII, HPFS	USA	210,700	/	Self-administered, FFQ-130, and validated	Tofu	CHD events (8359)	≥1 serving/week vs. <1 serving/month	a, b, and c	6
Ding (2016) [33]	NHS, NHSII, HPFS	USA	163,457	/	Self-administered, FFQ-116, and validated	Total soy food	T2D (9185)	≥1 serving/week vs. non-consumer	a, b, and c	6
Nanri (2010) [38]	JPHC I, II	Japan	59,791	10	Self-administered, FFQ-147, and validated	Soy products	T2D (1114)	Q5 vs. Q1 ≥ 186 vs. 29 g/d	a, b, and c	9
Yu (2015) [54]	SWHS	China	66,832	10	Interview, FFQ-77, and validated	Total soy foods (dry weight)	IS (3110)	Q5 vs. Q1 33.5 vs. 6.6 g/d	a, b, and c	9
Im (2021) [42]	KGES	Korea	4713	7.4	Interview, FFQ, and validated	Total soy foods	CVDs events (282)	Q4 vs. Q1 18.03 vs. 5.27 servings/week	a, b, and c	8
Katagiri (2019) [43]	JPHC	Japan	92,915	14.8	Self-administered, FFQ-138, and validated	Total soy products	CVDs deaths (3326) and stroke (1326)	Q5 vs. Q1 178 vs. 37 g/d	a, b, and c	9
Kobayashi (2019) [44]	JPHC	Japan	79,904	14.9	Self-administered, FFQ-138, and validated	Soy dietary diversity	CVDs deaths (2942)	Q5 vs. Q1 3.5 vs. 1.1 of food items/day	a, b, and c	9
Kokubo (2007) [45]	JPHC I	Japan	40,462	12.5	Self-administered, FFQ-44, and NA	Soy	stroke (1230) and MI (308)	Highest vs. lowest ≥ 5 vs. 0–2 days/week	a, b, and c	8
Konishi (2019) [36]	Takayama study	Japan	13,521	10	Self-administered, FFQ-169, and validated	Total soy foods	T2D (438)	Q3 vs. Q1 141.2 vs. 50.7 g/d	a, b, and c	8
Nguyen (2018) [49]	NIP-PON DATA	Japan	9244	24	Weigh and record all foods and beverages on 3 consecutive days	Tofu	stroke (417)	Q4 vs. Q1 41.5 vs. 0.8 g/1000 kcal	a, b, and c	8
Mueller (2011) [37]	SCHS	China	43,176	5.7	Interview, FFQ-165, and validated	Unsweetened soy	T2D (2252)	≥5/week vs. none	a, b, and c	8
*Case-control studies*				Baseline years ^a^						
Guo (2013) [40]	ICS	China	3547	1999.2–2003.3	Self-reported, FFQ-19, and validated	Tofu	CHD events (1312)	>3 vs. <0.75 times/week	a and b	6
Liang (2009) [46]	/	China	838	2007–2008	Interview, FFQ-125, and validated	Total soy foods	IS (374)	≥300 vs. <50 g	a, b, and c	7
Okamoto (2006) [56]	/	Japan	411	1992.4–1997.3	Interview, FFQ, and NA	Soy products	SAH (201)	Q4 vs. Q1	a and b	8
Fang (2006) [39]	/	USA	391	2000.9–2003.6	Interview, FFQ-49, and validated	Soybean	stroke (187)	≥3 vs. <3 times/week	a	5
Ho (2006) [41]	/	China	32,462	1997.12–1999.01	Proxy report, FFQ, and NA	Soy	stroke (2160) and fatal IHD (2016)	4+/week vs. <1/mon	a, b, and c	6
Sasazuki (2001) [55]	FHS	Japan	1846	1996.9–1998.9	Interview, FFQ-23, and NA	Tofu	AMI (632)	4+/week vs. <2/week	a and b	8
Sasazuki (2001) [55]	FHS	Japan	1846	1996.9–1998.9	Interview, FFQ-23, and NA	Miso soup	AMI (632)	2+/week vs. <1/week	b and c	8
Nguyen (2017) [32]	/	Vietnam	1198	2013.8–2015.10	Interview, FFQ-128, and validated	Total soy foods	T2D (599)	>133.9 vs. = <44.1 g/d	a, b, and c	7

Abbreviations: CVDs: cardiovascular diseases; CHD: coronary heart disease; IS: ischemic stroke; IHD: ischemic heart disease; SAH: subarachnoid hemorrhage; AMI: acute myocardial infarction; MI: myocardial infarction; T2D: type 2 diabetes; FFQ: Food Frequency Questionnaire; ICD: international classification of disease; JMS: Jichi Medical School Study; SWHS: Shanghai Women’s Health Study; JACC Study: Japan Collaborative Cohort Study for Evaluation of Cancer Risk; MR Cohort: Korean Multi-Rural Communities Cohort; CKB study: China Kadoorie Biobank study; SCHS: Singapore Chinese Health Study; JPHC: Japan Public Health Center-Based Study; JPHC I: Japan Public Health Center-Based Study Cohort I; JPHC I,II: Japan Public Health Center-Based Study Cohort I and Cohort II; ICS: Isfahan cohort study; NHS: Nurses’ Health Study; NHSII: Nurses’ Health Study II; HPFS: Health Professionals Follow-Up Study; KGES: Korean Genome and Epidemiology Study; NIP-PON DATA: National Integrated Project for Prospective Observation of Non-communicable Disease and its Trends in the Aged; ICS: INTERHEART China study; FHS: Fukuoka Heart Study; ADA: American Diabetes Association; WHO: World Health Organization; ECG: electrocardiogram; NA: not available; NOS: the Newcastle–Ottawa Scale; M: men; Mon: month; g: grams; g/d: grams/day; Q: quartile; y: years; Wang-a: individuals free of cardiovascular diseases at study baseline; and Wang-b: individuals with a history of cardiovascular diseases at study baseline. Degrees of adjustment for confounders were as follows: model a: sociodemographics, model b: risk factors for CVDs, and model c: dietary variables and other risk factors. ^a^ Baseline years were defined as included in the study analyzed.

**Table 2:** Stratified analyses between soy foods intake and the risk of T2D and CVDs events.

T2D Subgroup	No.	TRR (95% CI)	P_h_	*I*^2^ (%)	P_d_
Non-fermented soy food	8	0.90 (0.81, 1.00)	<0.001	77	0.05
Tofu	7	0.91 (0.81,1.02)	0.09	46	0.1
Soy milk	6	0.85 (0.68,1.05)	<0.001	90	0.12
Boiled beans	2	0.97 (0.74,1.26)	0.28	13	0.79
Others	1	1.04 (0.86,1.26)	0.38	0	0.66
Fermented soy food	2	0.98 (0.71,1.34)	0.68	0	0.89
Miso soup	2	0.98 (0.71,1.34)	0.68	0	0.89
Other soy food	5	0.75 (0.61,0.93)	<0.001	82	0.009
Subgroup total		0.87 (0.80,0.95)	<0.001	75	0.002
**CVDs Subgroup**					
Non-fermented soy food	16	0.92 (0.84,1.00)	<0.001	69	0.06
Tofu	14	0.89 (0.80,0.99)	<0.001	65	0.03
Soy milk	6	0.81 (0.59,1.12)	<0.001	77	0.2
Fermented soy food	10	0.86 (0.79,0.93)	0.08	41	<0.001
Natto	5	0.82 (0.75,0.90)	0.2	34	<0.001
Miso soup	8	0.93 (0.84,1.03)	0.12	39	0.17
Subgroup total		0.88 (0.83,0.93)	<0.001	66	<0.001
**CHD Subgroup**					
Non-fermented soy food	9	0.81 (0.70,0.95)	0.002	68	0.009
Tofu	7	0.81 (0.67,0.98)	0.003	70	0.03
Soy milk	3	0.80 (0.54,1.20)	0.10	57	0.28
Fermented soy food	7	0.85 (0.73,1.00)	0.65	0	0.05
Natto	3	0.89 (0.68,1.15)	0.26	26	0.36
Miso soup	6	0.96 (0.81,1.14)	0.78	0	0.63
Subgroup total		0.87 (0.80,0.94)	0.09	28	<0.001
**Stroke Subgroup**					
Non-fermented soy food	9	1.05 (0.97,1.13)	0.19	29	0.25
Tofu	7	1.01 (0.90,1.13)	0.15	36	0.89
Soy milk	2	0.50 (0.08,3.09)	<0.001	91	0.45
Fermented soy food	7	0.88 (0.78,1.00)	0.007	66	0.04
Natto	5	0.81 (0.72,0.91)	0.19	34	<0.001
Miso soup	6	0.91 (0.79,1.06)	0.04	57	0.23
Subgroup total		0.90 (0.84,0.97)	<0.001	69	0.005

Abbreviations: T2D: type 2 diabetes; CVDs: cardiovascular diseases; CHD: coronary heart disease; No: number; TRR: total relative risk; P_h_: *p* value for heterogeneity; and P_d_: *p* value for difference.

## Data Availability

Not applicable.

## Supplementary Materials

The following supporting information can be downloaded at: https://www.mdpi.com/article/10.3390/nu15061358/s1, Table S1: Detailed search strategies for Pubmed, Embase, Medline, and Web of Science by Ovid SP, Table S2: The pooled risk association of stroke with the consumption of soy foods, Table S3(A): The Newcastle–Ottawa Scale criteria for quality of cohort studies, Table S3(B): The Newcastle–Ottawa Scale criteria for quality of case–control studies, Table S4: Stratified analyses between baseline characteristics and the risk of T2D and CVDs events, Figure S1: The pooled risk association of coronary heart disease with the consumption of soy foods, Figure S2: The pooled risk association of type 2 diabetes and cardiovascular diseases with the consumption of soy protein, Figure S3: Dose–response analysis for the potential curvilinear or linear associations of soy intake with cardiovascular diseases, coronary heart disease, and stroke, Figure S4: Begg’s funnel plot of risk association of (A) cohort studies of cardiovascular diseases, (B) case–control studies of cardiovascular diseases, (C) type 2 diabetes with the consumption of soy foods, Figure S5: Sensitivity analysis of risk association of type 2 diabetes (A) and cardiovascular diseases (B). References [84,85,86,87,88,89,90,91,92,93,94,95,96,97,98] are cited in the supplementary materials.
