# Supplementary material for: Soy Consumption and the Risk of Type 2 Diabetes and Cardiovascular Diseases: A Systematic Review and Meta-Analysis
Source: Nutrients. 2023 Mar 10;15(6):1358. doi: 10.3390/nu15061358 (PMC10058927; doi:10.3390/nu15061358)

Soy consumption and the risk of type 2 diabetes and cardiovascular diseases: a systematic review and meta-analysis

Table S1 Detailed search strategies for Pubmed, Embase, Medline, and Web of Science by Ovid SP

1	Soybean Proteins.mp. or exp Soybean Proteins
2	Soybeans.mp. or exp Soybeans
3	soy foods.mp. or exp Soy Foods
4	("Soybean Protein*" or "Soy Bean Protein*" or "Dietary Soybean Protein*" or "Protein*, Dietary Soybean" or "Soybean Protein*, Dietary" or "Soy Protein*" or "Protein*, Soy" or "Protein*, Soybean" or "Soybean*" or "Soy Bean*" or "Bean*, Soy" or "soya bean" or "Soy Food*" or "Tofu" or "miso" or "sufu" or "soymilk" or "Natto" or "soy sauce" or "soy bean curd*" or "soy cheese*" or "Tempeh" or "Texturized Soy Protein*").ab,ti.
5	1 or 2 or 3 or 4
6	Diabetes Mellitus.mp. or exp Diabetes Mellitus
7	("Diet, Diabetic" or diabetes or "Diabetes Mellitus" or "DM").ab,ti.
8	6 or 7
9	Prospective Studies.mp. or Prospective Studies
10	Cohort Studies.mp. or Cohort Studies
11	Longitudinal Studies.mp. or Longitudinal Studies
12	(prospective or longitudinal or cohort or cohorts or "follow-up" or "case-cohort" or "nested case-control").ab,ti.
13	9 or 10 or 11 or 12
14	4 and 8 and 13
15	limit 14 to humans
16	Coronary Disease.mp. or exp Coronary Disease
17	Coronary Artery Disease.mp. or exp Coronary Artery Disease
18	Myocardial Ischemia.mp. or exp Myocardial Ischemia
19	Myocardial Infarction.mp. or exp Myocardial Infarction
20	Acute Coronary Syndrome.mp. or exp Acute Coronary Syndrome
21	Angina Pectoris.mp. or exp Angina Pectoris

Table S1 Detailed search strategies for Pubmed, Embase, Medline, and Web of Science by Ovid SP (continued)

22	Heart Diseases.mp. or exp Heart Diseases
23	Cardiovascular Diseases.mp. or exp Cardiovascular Diseases
24	(Coronary Disease* or Disease*, Coronary or Coronary Heart Disease* or Disease*, Coronary Heart or "CHD" or "CAD" or Coronary Artery Disease* or Artery Disease*, Coronary or Left Main Coronary Artery Disease* or Left Main Disease* or Coronary Arteriosclerosis or Coronary Atherosclerosis or Coronary Arterioscleroses or Myocardial Ischemia* or Ischemic Heart Disease* or Ischemia*, Myocardial or Heart Disease*, Ischemic or myocardial infarction* or Myocardial Infarct* or Heart Attack* or Cardiovascular Stroke* or acute coronary syndrome* or Angina Pectoris or Angor Pectoris or angina or Stenocardia* or unstable angina or heart disease* or Cardiac Disease* or Cardiac Disorder* or Heart Disorder* or Cardiovascular Disease*).ab,ti.
25	16 or 17 or 18 or 19 or 20 or 21 or 22 or 23 or 24
26	5 and 13 and 25
27	limit 26 to humans
28	exp Stroke/ or Stroke.mp.
29	(ischemic or ischaemic or hemorrhagic or haemorrhagic).ab,ti.
30	"stroke*".ab,ti.
31	28 or 30
32	29 and 31
33	(Cerebrovascular Accident* or Cerebrovascular Apoplexy or Brain Vascular Accident* or Cerebrovascular Stroke* or Apoplexy or Cerebral Stroke* or Cerebrovascular Disorder* or cerebral infarction* or cerebral hemorrhage or cerebral haemorrhage or Acute Cerebrovascular Accident* or Acute Stroke*).ab,ti.
34	32 or 33
35	5 and 13 and 34
36	limit 35 to humans

Table S2 Fifteen articles were further excluded based on the inclusion and exclusion criteria

Study	Reason for the exclusion
Lukaszuk [84]	Study design did not meet the inclusion criteria
Menotti [85]	Exposure of interest did not meet inclusion criteria
Nagura [86]	Exposure of interest did not meet inclusion criteria
Nanri [87]	Exposure of interest did not meet inclusion criteria
Ozawa [88]	Outcome of interest did not meet the inclusion criteria
Preis [89]	Exposure of interest did not meet inclusion criteria
Tsugane [90]	Outcome of interest did not meet the inclusion criteria
Yu [91]	letter
Morimoto [92]	Short communication
Yang [93]	Duplicate data
Nagata [47]	Duplicate data
Kokubo [94]	Meeting abstract
Tavani [95]	Exposure of interest did not meet inclusion criteria
Mink [96]	Exposure of interest did not meet inclusion criteria
Mccullough [97]	Exposure of interest did not meet inclusion criteria
Loke [98]	Outcome of interest did not meet the inclusion criteria

Table S3 (A) The Newcastle-Ottawa Scale criteria for quality of cohort studies

Study	Representativeness of the exposed cohort	Selection of the non-exposed cohort	Ascertainment of exposure	Demonstration that outcome of interest was not present at the start of the study	Comparability of cohorts based on the design or analysis	Assessment of outcome	Was follow-up long enough for outcomes to occur	Adequacy of follow-up of cohorts	Total quality scores
Yamasaki (2015)	☆	☆	☆	☆	☆☆	☆	☆	/	8
Nguyen (2016)	☆	☆	☆	☆	☆☆	☆	☆	/	8
Nagata (2016)	☆	☆	☆	☆	☆☆	☆	☆	☆	9
Zhang (2003)	☆	☆	☆	☆	☆☆	☆	☆	/	8
Yan (2021)	☆	☆	☆	☆	☆☆	☆	☆	☆	9
Woo (2020)	☆	☆	☆	☆	☆☆	☆	☆	☆	9
Wang (2021)	☆	☆	☆	/	☆☆	☆	☆	/	7
Villegas (2008)	☆	☆	☆	☆	☆☆	☆	☆	☆	9
Tatsumi (2013)	☆	☆	☆	☆	☆	☆	☆	/	7
Talaei (2014)	☆	☆	☆	☆	☆☆	☆	☆	☆	9
Nozue (2020)	☆	☆	☆	☆	☆☆	☆	☆	/	8
Nouri (2021)	☆	☆	☆	☆	☆	☆	☆	/	7
Ma (2020)	/	/	☆	☆	☆☆	☆	☆	/	6
Ding (2016)	/	/	☆	☆	☆☆	☆	☆	/	6
Nanri (2010)	☆	☆	☆	☆	☆☆	☆	☆	☆	9
Yu (2015)	☆	☆	☆	☆	☆☆	☆	☆	☆	9
Im (2021)	☆	☆	☆	☆	☆	☆	☆	☆	8
Katagiri (2019)	☆	☆	☆	☆	☆☆	☆	☆	☆	9
Kobayashi (2019)	☆	☆	☆	☆	☆☆	☆	☆	☆	9

Table S3 (A) The Newcastle-Ottawa Scale criteria for quality of cohort studies (continued)

Study	Representativeness of the exposed cohort	Selection of the non-exposed cohort	Ascertainment of exposure	Demonstration that outcome of interest was not present at the start of the study	Comparability of cohorts based on the design or analysis	Assessment of outcome	Was follow-up long enough for outcomes to occur	Adequacy of follow-up of cohorts	Total quality scores
Kokubo (2007)	☆	☆	☆	☆	☆☆	☆	☆	/	8
Konishi (2019)	☆	☆	☆	☆	☆☆	☆	☆	/	8
Mueller (2011)	☆	☆	☆	☆	☆☆	☆	☆	/	8

Table S3 (B) The Newcastle-Ottawa Scale criteria for quality of case-control studies

Study	Is the Case Definition Adequate?	Representativeness of the Cases	Selection of Controls	Definition of Controls	Comparability of Cases and Controls on the Basis of the Design or Analysis	Ascertainment of Exposure	Same method of ascertain-ent for cases and controls	Non-Response Rate	Total quality scores
Guo (2013)	☆	☆	/	☆	☆☆	☆	/	/	6
Liang (2009)	☆	☆	/	☆	☆☆	☆	☆	/	7
Okamoto (2006)	☆	☆	☆	☆	☆☆	☆	☆	/	8
Fang (2006)	☆	☆	/	☆	☆	☆	/	/	5
Ho (2006)	☆	☆	/	☆	☆☆	☆	/	/	6
Sasazuki (2001)	☆	☆	☆	☆	☆☆	☆	☆	/	8
Nguyen (2017)	☆	☆	/	☆	☆☆	☆	☆	/	7

Table S4 Stratified analyses between baseline characteristics and the risk of type 2 diabetes (T2D) and cardiovascular diseases (CVDs) events

**T2D Subgroup**	No.	TRR (95% CI)	P_h	I^2(%)	P_d	**CVDs subgroup**	No.	TRR (95% CI)	P_h	I^2(%)	P_d
Study design						Study design					
Case-control	1	0.31 (0.21, 0.46)	NA	NA	<0.001	Case-control	8	0.62 (0.49, 0.79)	0.001	71	<0.001
Cohort	14	0.88 (0.80, 0.96)	<0.001	68	0.005	Cohort	23	0.93 (0.87, 0.99)	<0.001	73	0.03
Subgroup total	15	0.83 (0.74, 0.93)	<0.001	80	0.002	Subgroup total	31	0.86 (0.80, 0.93)	<0.001	78	<0.001
Sex						Sex					
Men	6	0.99 (0.89,1.10)	0.46	0	0.81	Men	9	0.96 (0.89,1.04)	0.17	31	0.37
Women	7	0.78 (0.67,0.91)	<0.001	82	0.001	Women	13	0.80 (0.68,0.94)	<0.001	84	0.005
Both	2	0.56 (0.18,1.70)	<0.001	96	0.3	Both	9	0.81 (0.70,0.94)	<0.001	85	0.005
Subgroup total	15	0.83 (0.74,0.94)	<0.001	80	0.002	Subgroup total	31	0.86 (0.80, 0.93)	<0.001	78	<0.001
Study quality						Study quality					
High	10	0.86 (0.73,1.00)	<0.001	74	0.05	High	20	0.92 (0.85,1.00)	<0.001	75	0.06
Moderate	5	0.79 (0.64,0.96)	<0.001	87	0.02	Moderate	11	0.76 (0.66,0.88)	<0.001	80	<0.001
Subgroup total	15	0.83 (0.74,0.93)	<0.001	80	0.002	Subgroup total	31	0.86 (0.80, 0.93)	<0.001	78	<0.001
Study region						Study region					
Asia	12	0.79(0.64,0.96)	<0.001	82	0.02	Asia	27	0.88 (0.81,0.95)	<0.001	80	0.001
USA	3	0.93(0.88,0.98)	0.38	0	0.008	USA	4	0.79 (0.64,0.97)	0.11	50	0.02
Subgroup total	15	0.83(0.74,0.94)	<0.001	80	0.002	Subgroup total	31	0.86 (0.80, 0.93)	<0.001	78	<0.001
Follow-up years						Follow-up years					
≥8 y	4	0.85 (0.65,1.11)	0.01	72	0.24	≥13 y	10	0.99 (0.94,1.05)	0.37	7	0.82
< 8 y	7	0.86 (0.70,1.06)	<0.001	75	0.15	< 13 y	9	0.91 (0.79,1.05)	<0.001	83	0.2
Subgroup total	11	0.85 (0.73,1.00)	<0.001	71	0.05	Subgroup total	19	0.96 (0.90, 1.03)	<0.001	68	0.25
**CHD Subgroup**	No.	TRR (95% CI)	P_h	I^2(%)	P_d	**Stroke subgroup**	No.	TRR (95% CI)	P_h	I^2(%)	P_d
Study design						Study design					
Case-control	5	0.69 (0.58,0.83)	0.22	30	<0.001	Case-control	5	0.57 (0.35,0.92)	<0.001	80	0.02

Table S4 Stratified analyses between baseline characteristics and the risk of T2D and CVDs events (continued)

**CHD Subgroup**	No.	TRR (95% CI)	P_h	I^2(%)	P_d	**Stroke subgroup**	No.	TRR (95% CI)	P_h	I^2(%)	P_d
Cohort	12	0.84 (0.74,0.94)	0.001	64	0.003	Cohort	13	0.96 (0.87,1.05)	<0.001	73	0.35
Subgroup total	17	0.79 (0.71, 0.88)	<0.001	65	<0.001	Subgroup total	18	0.88 (0.79, 0.99)	<0.001	80	0.03
Sex						Sex					
Men	6	0.85 (0.75,0.95)	0.18	34	0.007	Men	7	0.98 (0.83, 1.16)	0.005	71	0.85
Women	7	0.63 (0.50,0.81)	0.02	62	<0.001	Women	6	0.88 (0.73, 1.06)	<0.001	83	0.19
Both	4	0.89 (0.75,1.06)	0.02	70	0.20	Both	5	0.71 (0.53, 0.95)	<0.001	85	0.02
Subgroup total	17	0.79 (0.71, 0.88)	<0.001	65	<0.001	Subgroup total	18	0.88 (0.79, 0.99)	<0.001	80	0.03
Study quality						Study quality					
High	9	0.81 (0.68,0.96)	<0.001	70	0.01	High	12	0.95 (0.85,1.06)	<0.001	76	0.33
Moderate	8	0.77 (0.68,0.89)	0.02	57	<0.001	Moderate	6	0.72 (0.53,0.97)	<0.001	83	0.03
Subgroup total	17	0.79 (0.71, 0.88)	<0.001	65	<0.001	Subgroup total	18	0.88 (0.79, 0.99)	<0.001	80	0.03
Study region						Study region					
Asia	14	0.79 (0.69,0.90)	<0.001	67	<0.001	Asia	17	0.89 (0.80, 0.99)	<0.001	81	0.04
USA	3	0.79 (0.62,1.01)	0.06	65	0.06	USA	1	0.72 (0.41, 1.26)	-	-	0.25
Subgroup total	17	0.79 (0.71, 0.88)	<0.001	65	<0.001	Subgroup total	18	0.88 (0.79, 0.99)	<0.001	80	0.03
Follow-up years						Follow-up years					
≥13 y	4	0.88 (0.74,1.05)	0.07	58	0.16	≥13 y	8	0.96 (0.85, 1.08)	0.005	66	0.47
< 13 y	5	0.79 (0.60,1.03)	0.003	75	0.09	< 13 y	5	0.95 (0.80,1.12)	<0.001	80	0.52
Subgroup total	9	0.85 (0.74, 0.98)	0.003	66	0.03	Subgroup total	13	0.96 (0.87, 1.05)	<0.001	73	0.35

Abbreviations: T2DM: type 2 diabetes; CVDs: cardiovascular diseases; CHD: coronary heart disease; No: numero; TRR: total relative risk; P_h: P value for heterogeneity; P_d: P value for differenc

Figure S1 The pooled risk association of CHD with the consumption of soy foods. CI: confidence interval

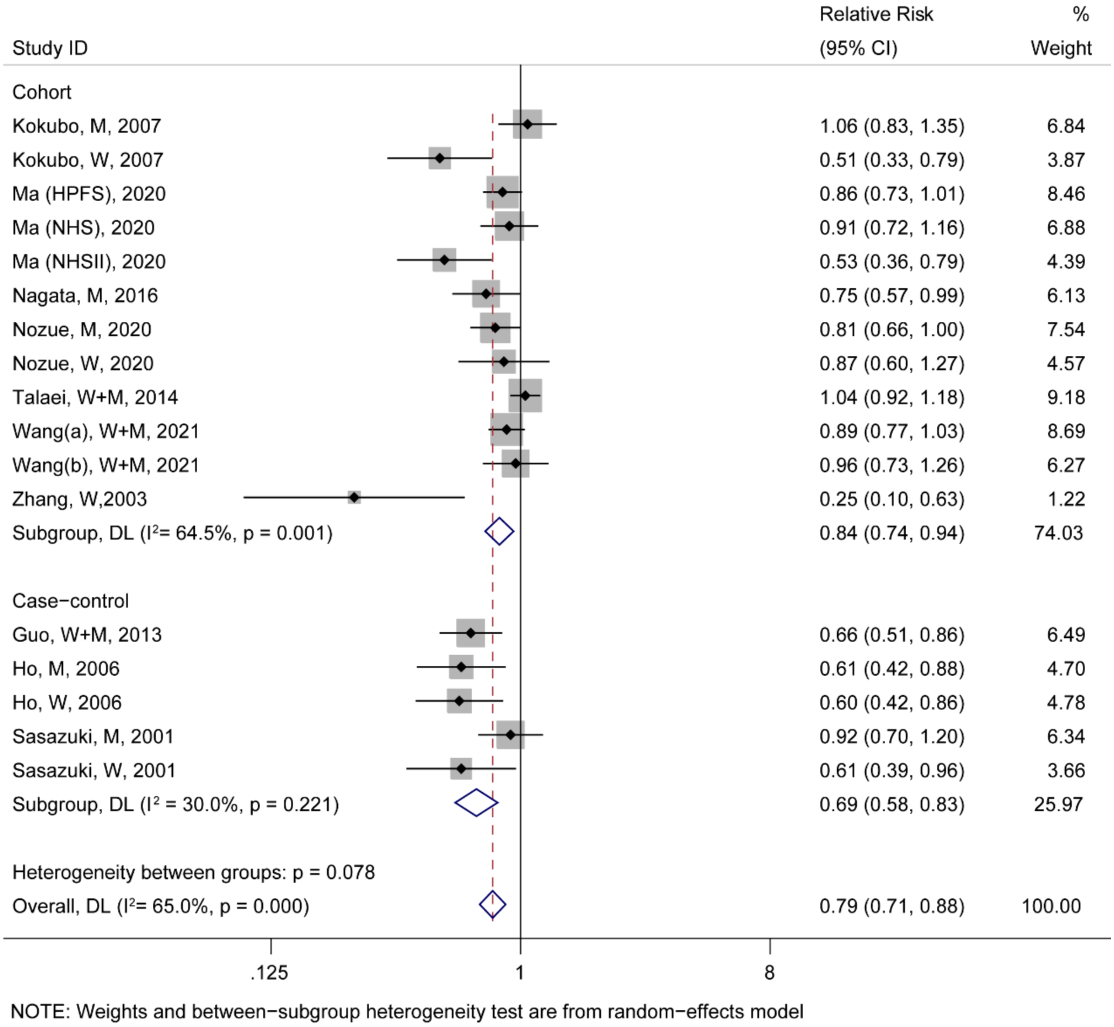

Figure S2 The pooled risk association of stroke with consumption of soy foods. CI: confidence interval

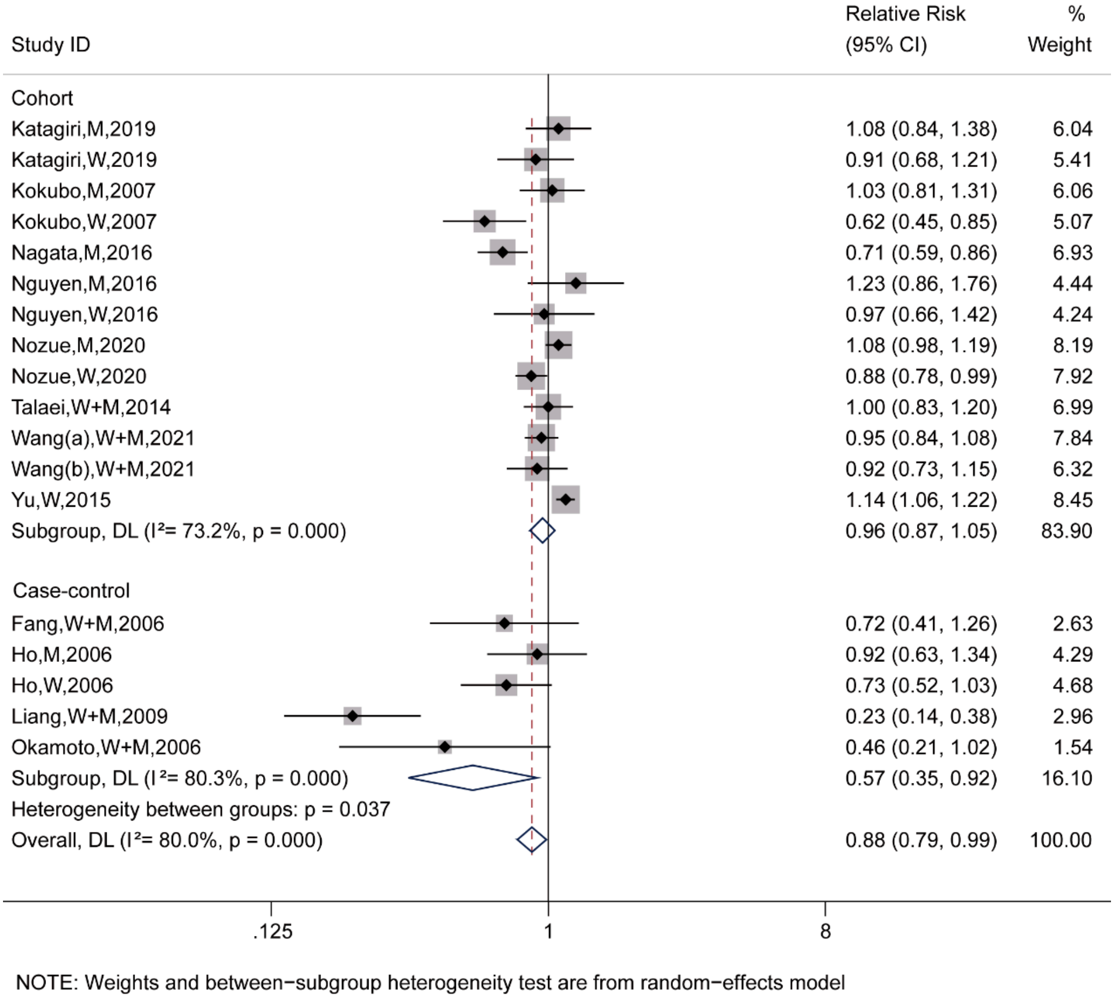

Figure S3 Dose-response analysis for the potential non-linear or linear associations of soy intake with CVDs, CHD, and stroke

（**A**） Tofu and CVDs

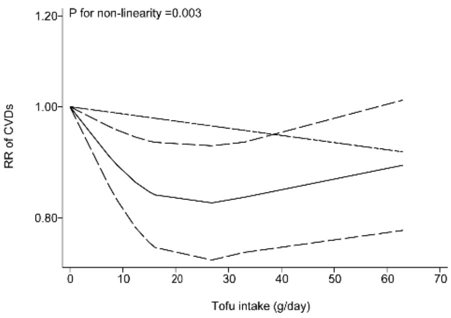

(**B**) Natto and CVDs

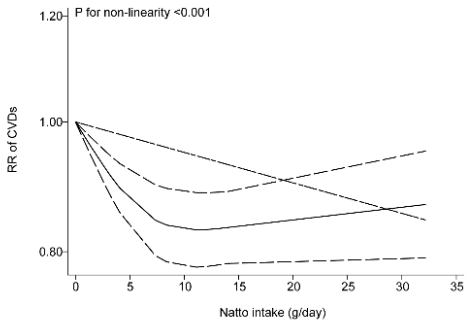

(**C**) Natto and stroke

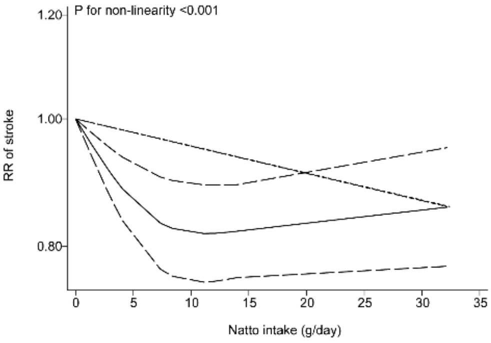

(**D**) Miso and CVDs

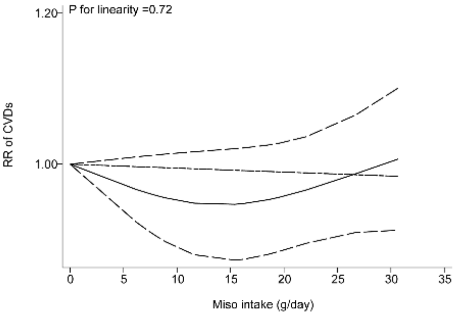

(**E**) Natto and CHD

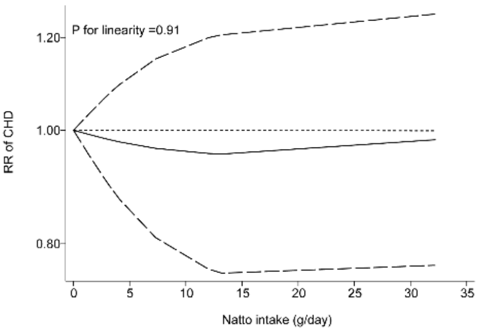

(**F**) Miso and stroke

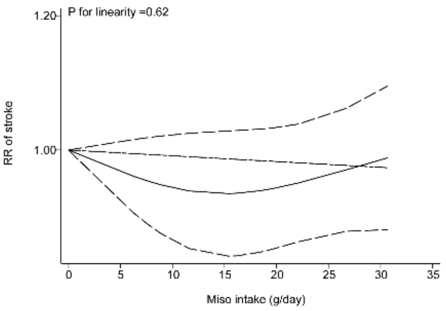

Figure S4 Begg's funnel plot of risk association of (**A**) cohort studies of CVDs, (**B**) C, and (**C**) T2D with the consumption of soy foods. LnRR: log of relative risk; S.E.: standard error

(A) Cohort studies of CVDs

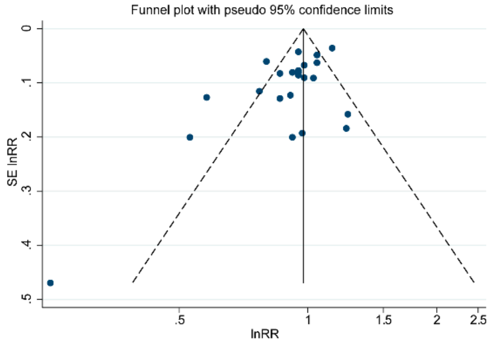

(B) Case-control studies of CVDs

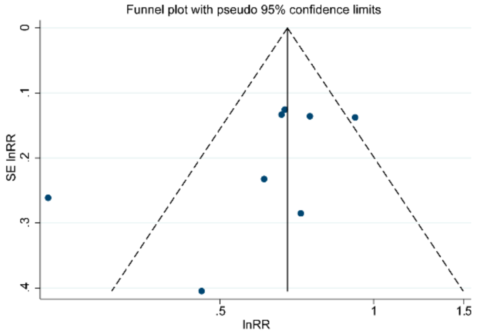

(C) Soy foods and T2D

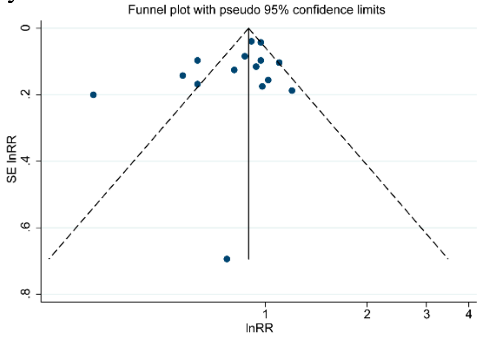

Figure S5 Sensitivity analysis of risk association of T2D (**A**) and CVDs (**B**)

(**A**)

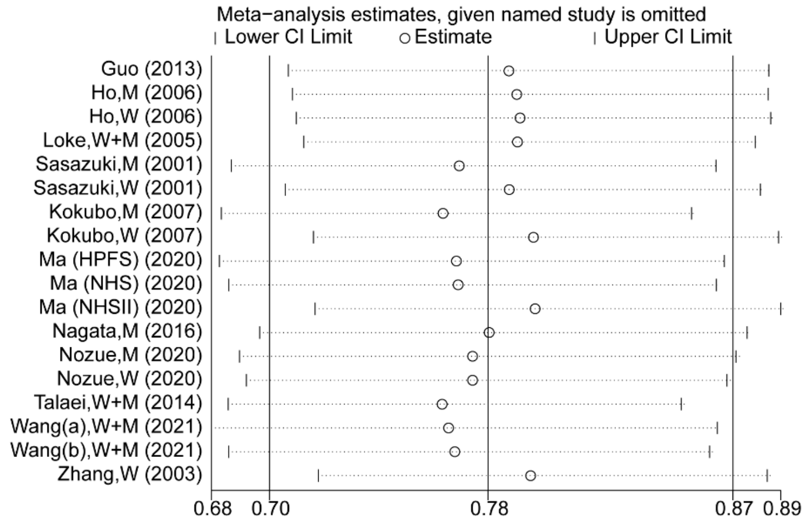

(**B**)

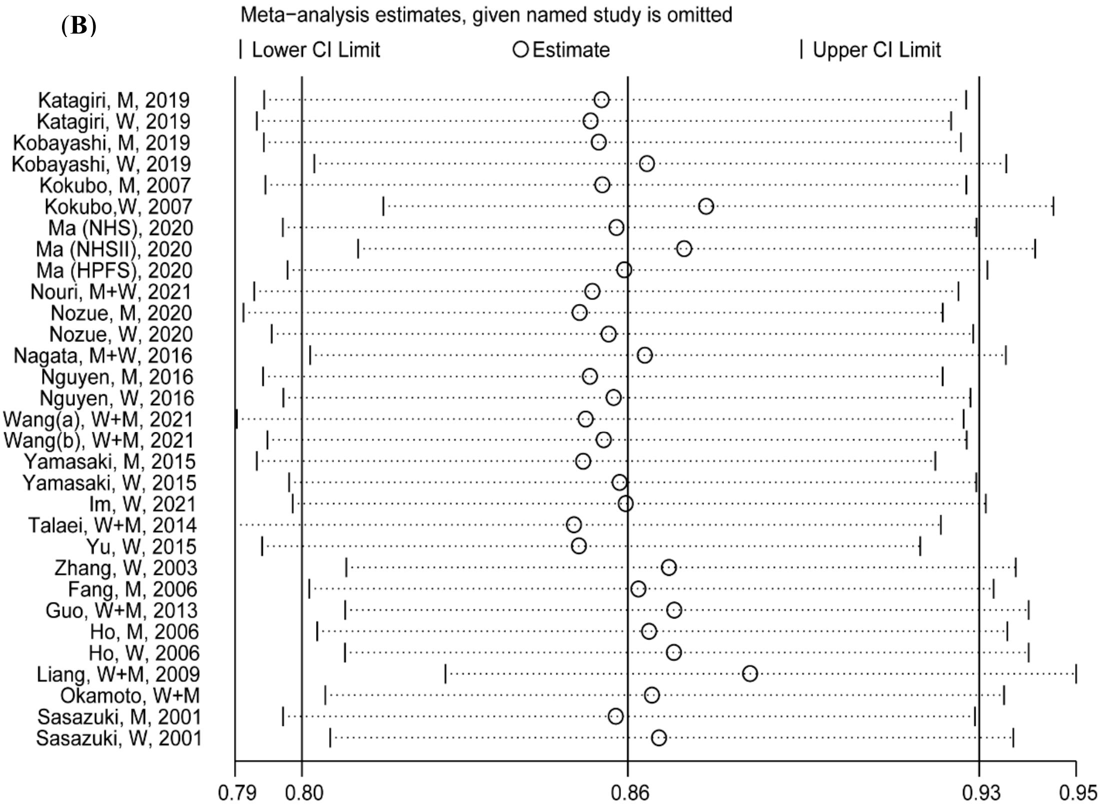

Supplement: Supplementary file 1 [file nutrients-15-01358-s001.zip › nutrients-2240871-Supplementary Files.pdf]
